# Modeling the ***Influenza A*** NP-vRNA-Polymerase Complex in Atomic Detail

**DOI:** 10.3390/biom11010124

**Published:** 2021-01-19

**Authors:** Jacob C. Miner, Anna Lappala, Paul W. Fenimore, William M. Fischer, Benjamin H. McMahon, Nicolas W. Hengartner, Karissa Y. Sanbonmatsu, Chang-Shung Tung

**Affiliations:** 1 Theoretical Biology and Biophysics Group, Theoretical Division, Los Alamos National Laboratory, Los Alamos, NM 87545, USA; jcminer@lanl.gov (J.C.M.); lappala@molbio.mgh.harvard.edu (A.L.); paulf@lanl.gov (P.W.F.); wfischer@lanl.gov (W.M.F.); mcmahon@lanl.gov (B.H.M.); nickh@lanl.gov (N.W.H.); kys@lanl.gov (K.Y.S.); 2 Bioenergy and Biome Sciences Group, Bioscience Division, Los Alamos National Laboratory, Los Alamos, NM 87545, USA; 3 Department of Molecular Biology & Massachusetts General Hospital, Harvard University, 185 Cambridge St., Boston, MA 02114, USA; 4 New Mexico Consortium, Los Alamos, NM 87545, USA

**Keywords:** *Influenza A*, RNP, viral genome, homology modeling

## Abstract

Seasonal flu is an acute respiratory disease that exacts a massive toll on human populations, healthcare systems and economies. The disease is caused by an enveloped *Influenza* virus containing eight ribonucleoprotein (RNP) complexes. Each RNP incorporates multiple copies of nucleoprotein (NP), a fragment of the viral genome (vRNA), and a viral RNA-dependent RNA polymerase (POL), and is responsible for packaging the viral genome and performing critical functions including replication and transcription. A complete model of an *Influenza* RNP in atomic detail can elucidate the structural basis for viral genome functions, and identify potential targets for viral therapeutics. In this work we construct a model of a complete *Influenza A* RNP complex in atomic detail using multiple sources of structural and sequence information and a series of homology-modeling techniques, including a motif-matching fragment assembly method. Our final model provides a rationale for experimentally-observed changes to viral polymerase activity in numerous mutational assays. Further, our model reveals specific interactions between the three primary structural components of the RNP, including potential targets for blocking POL-binding to the NP-vRNA complex. The methods developed in this work open the possibility of elucidating other functionally-relevant atomic-scale interactions in additional RNP structures and other biomolecular complexes.

## 1. Introduction

Influenza is a respiratory illness caused by *Influenza* viruses from the *Orthomyxoviridae* family, which are in continuous circulation around the world [1]. Despite the biannual development of new vaccines, influenza-related mortality estimates are as high 650,000 per annum [2], making new therapeutics a priority for mitigating the global impact of influenza. The *Influenza A* virus, which is responsible for most seasonal cases, and all historical epidemics, will serve as the focus of this project. *Influenza A* possesses a negative-strand RNA genome that is fragmented into eight segments within the *Influenza A* virus. In each segment, multiple copies of nucleoprotein (NP) form a macromolecular structure that compactifies a section of the viral RNA (vRNA) to form a ribonucleoprotein complex (RNP) [3,4]. Each RNP is in turn complexed with its own RNA-dependent RNA polymerase (POL). Taken together, these complexes contribute to many functional roles in the *Influenza A* virus including (i) replication and transcription of the viral genome, (ii) intracellular transport, (iii) gene re-assortment, (iv) viral genome packaging into progeny particles, and (v) host adaptation [5,6]. In order to develop a better understanding of how RNP complexes perform these critical functions, and gain insight into how these functions might be tuned and controlled, the atomic structure of the complete RNP complex is essential.

Models of large molecular complexes provide valuable insight into structure-based molecular mechanics and allow for the identification of potential target sites for drug-binding. Macromolecular complexes can be generated with sufficient accuracy to accomplish these goals using the vast amount of sequence and structure information collected by the biosciences community [7,8,9,10], and with directed homology modeling methods such as motif-matching fragment assembly (MMFA). Our group has employed MMFA methods to generate models of *T. thermophilus* ribosome [11,12], ribosomal subunits of *E. coli* [13], viral envelope proteins from Zika and Ebola viruses [14], and *E. coli* transcription initiation complexes [15]. In each of these studies, we modeled the complexes at atomic precision and predicted interactions responsible for functional behaviors. These successes have been made possible by leveraging the vast amount of structural data for proteins and nucleic acids provided by the Protein Data Bank (PDB) [8,9] and Electron Microscopy Data Bank (EMDB) [10]. Importantly, we employ structural information with different resolutions and levels of completion in order to generate complete macromolecular models.

By applying these techniques to the RNP complex of *Influenza A* we can use atomic-scale fragments from the PDB, and coarse-grain envelopes from EMDB, to generate a complete RNP model with atomic-scale accuracy, marking a critical advance to previous studies of RNPs by elucidating more precise structural interactions within these complexes [16,17,18]. For relevance to human diseases, we model this RNP complex using a sequence of *Influenza A* adapted to infect humans (A/Albany/3/1967) [19]. Our predicted model involves (i) generating a vRNA genome of over 800 residues in atomic detail based on phosphorous atom coordinate information [3], (ii) accurately orienting NP folded domains based on NP-NP oligomers from helical and circular suprastructures [20,21,22,23,24,25,26,27], (iii) docking a polymerase derived from bat *Influenza A* [28] to the RNP in a way that will permit vRNA transcription and translation [3,29,30,31,32], and (iv) merging all of these components into a single macromolecular complex that satisfies the hairpin structure of RNP. After assembling the RNP, a gas-phase energy minimization resolves atomic-scale clashes within the complex and identifies the most salient salt-bridge interactions. Based on this model we are able to provide a structure–function explanation for reduced POL-activity in in vitro studies of mutated NP particles [33]. Lastly, we discuss how additional experiments can be validated by our model.

With infectious diseases exacting an increasingly ruinous toll on human populations and economies, the value of being able to identify the structure-function dependencies of biomolecular complexes in pathogens, and identify where novel therapeutics might best affect their pathogenic function, is greater than ever [34]. Over 156,000 biomolecular structures have been determined and deposited in the current version of Protein Data Bank [9] after decades of concerted efforts. However, only ∼5% (<8000) of all structures available in the PDB are characterized as nucleic-acid/protein complexes. Our modeling approach has the potential to model other nucleic-acid/protein complexes and genomic fragments at different functional stages in order to validate experimental results, and to direct the development of new therapeutics against viral infections.

## 2. Materials and Methods

### 2.1. Modeling of the vRNA Phosphate-Ribose Backbone

We defined each nucleotide along the length of the vRNA by two torsional angles: α and β. The α angle corresponds to the backbone orientation (P${}_{i-1}-$P${}_{i}-$P${}_{i+1}-$P${}_{i+2}$), while β corresponds to the position of the nucleobase (P${}_{i-1}-$P${}_{i}-$P${}_{i+1}-$C1′${}_{i}$), where the subscript *i* represents the nucleotide of interest. In order to provide a general representation of the range of the known structure space of RNA in the PDB we use the crystal structure of *T. thermophilus* 30S rRNA (PDB accession code: 1J5E). This allows for a correlation between α and β torsions to be determined, and this information allows for the orientation of nucleobases of the vRNA along the P-atom trace as part of generating an initial vRNA model.

### 2.2. Homology Modeling Using a Motif-Matching Fragment Assembly Method (MMFA)

The MMFA described in our earlier work [13] is used to conduct homology modeling of the ribonucleoprotein (RNP) complex and the polymerase (POL) of *Influenza*. This approach allows the full RNP complex to be assembled using partial structures of proteins and RNA at different resolutions from both X-ray crystallography and cryo-electron microscopy (cryo-EM) data sources.

### 2.3. Structure Alignment and Superposition

Structural assembly of the RNP hairpin is accomplished by superposition of individual NPs from circular and helical suprastructures. The MatchMaking option in the Structural Comparison Tool of Chimera [35] allows for structural alignment and superposition of the structurally-matched segments.

### 2.4. Modeling of Protein Loop Structure

Using 62 high-resolution (1 Å or better) non-homologous protein structures from PDB as structural references, we identified short loops with the same sequence length as our targets, and match the loop ends to the ends of the template protein. A target threshold of 0.1 Å RMSD is used to find matching main-chain atoms of the templates and targets. Out of our set of potential candidates, we further eliminate those which have bad contacts with the neighboring residues of the loop.

### 2.5. Structural Refinement

To resolve any bad contacts in our RNP model we employ a pairwise-distance calculator to check contacts between individual proteins (NP or POL) with one another, and between individual proteins and the vRNA. Resolving close contacts and the most extreme van der Waals overlaps (typically within 1 Å) is done by regenerating side-chains of clashing residues in different orientations that do not clash.

Lastly, we perform energy minimization and microcanonical ensemble (NVE) simulations using the GROMACS software package (ver. 4.5.5) [36] with the AMBER99 force field [37] and Chen-Garcia modified RNA parameters [38]. This force field was chosen for its ability to stabilize a variety of RNA conformations [39], and for its prior use in protein-RNA characterization [40]. A 1000-step, steepest-descent energy minimization (EM) resolves initial clashes and bad contacts in our RNP model. The output of this EM serves as the initial state for a 100-picosecond NVE simulation (50,000, 2-femtosecond time-steps) in gas-phase using a leap-frog integrator and particle velocities generated from a 300 K Maxwell distribution. A follow-up 1000-step steepest-descent EM of the last frame of the NVE simulation produces the final RNP model. In all simulations, heavy atoms of the RNP model (protein C${}_{\alpha }$ atoms and RNA P atoms) are given position restraints of 1000 kJ·mol${}^{-1}\cdot$nm${}^{-2}$ to prevent deformation of the overall RNP architecture and allow side-chains and nucleobases to relax into their optimal arrangements.

The goodness-of-fit for select components of our model is calculated using the Fit-to-Map utility in Chimera [35].

### 2.6. Graphics

Molecular graphics images in this work are produced using the UCSF Chimera package [41] from the Resource for Bio-computing, Visualization and Informatics at the University of California, San Francisco.

## 3. Results

Our modeling approach utilizes multiple types of sequence and structural information, and we model each RNP component using different information sources (Table 1). As we generate the atomic-scale fragments of each supramolecular structure, any bad contacts formed within a single monomeric unit (i.e., vRNA atoms clashing with NP atoms) are fixed by repeating the same adjustments on all other monomeric units within the same suprastructure. In this way, despite each RNP target depending on the immediate data sources listed in Table 1, every component is refined using input from the other components that make up its nearest neighbors in the final macromolecular assembly. This ensures self-consistency in the final macromolecular model.

### 3.1. Modeling of RNA and Nucleoprotein

We determine initial models for vRNA and nucleoprotein (NP), and orient them relative to one another using electron microscopy envelopes described by experimental data.

#### 3.1.1. All-Atom Model of a Single-Stranded RNA from a Low-Resolution Structure

The backbone of the single-stranded vRNA provides the primary point of contact with the RNA-binding grooves of each NP. This allows us to insert a genome segment of the *Influenza* genome using only the phosphorous atom trace to guide placement [16,21]. Using atomic-scale, single-stranded RNA fragments from the PDB we can match the phosphorous atoms to the trace and fill in the 3D coordinates of the rest of the vRNA. Like all RNA molecules, the single-stranded RNA genome of *Influenza* carries a large sum of negative charges in its phosphate backbone. Packaging of this molecule is only possible due to the many positively-charged residues in the RNA-binding groove on the surface of NP. This encapsidation is critical to transcription, replication and viral packaging [25].

**Figure 1:**
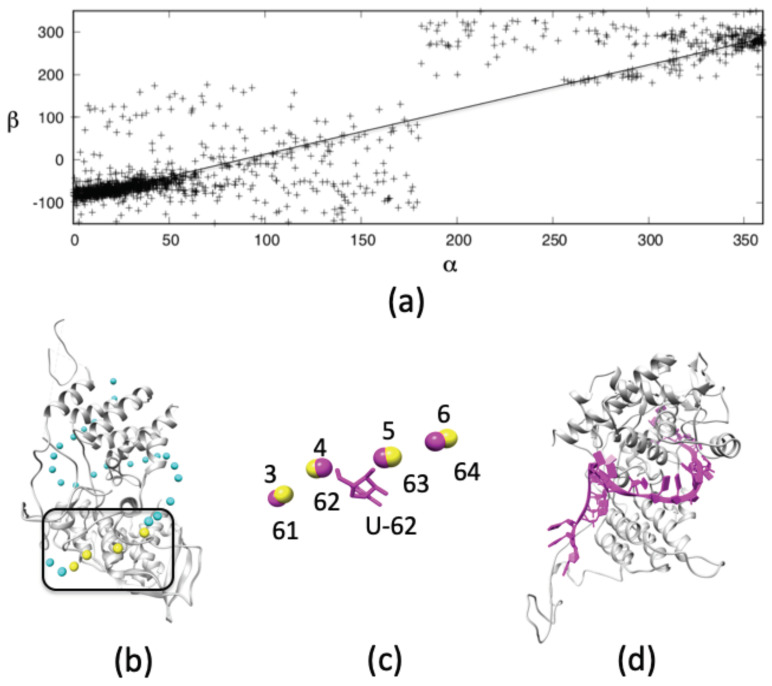
Single-stranded RNA structure prediction. (**a**) The α and β torsional angles for each nucleotide in *T. thermophilus* 30S rRNA shows a linear correlation, allowing an initial prediction of nucleobase orientation based on the backbone torsion. (**b**) Four target phosphorous atoms (in yellow) of vRNA at the NP surface align to the phosphorous atoms of a template structure ((**c**), in magenta), from which the embedded nucleoside structure is derived. (**d**) By repeating this method across the NP surface a model of the vRNA is generated.

In order to determine the orientation of the nucleobases of vRNA around NP we need to determine how the orientation of the sugar-base (β: P${}_{i-1}$-P${}_{i}-$P${}_{i+1}-$C1′${}_{i}$) correlates with the local structure of the phosphate backbone (α: P${}_{i-1}$-P${}_{i}$-P${}_{i+1}$-P${}_{i+2}$), where the subscript *i* represents the nucleotide of interest. We calculate α and β torsional angles using the full length of the 30S rRNA of *T. thermophilus* (PDB accession code: 1J5E) and observe a linear correlation between both angles (Figure 1a); slope and intercept corresponding to 0.97 and –82.6${}^{\circ }$, respectively. This correlation of backbone geometry to nucleobase orientation permits the phosphorous coordinates to serve as a template for orienting the nucleobases of the vRNA.

In Figure 1b we demonstrate how four consecutive phosphorous atoms of vRNA on the surface of NP (PDB accession code: 4BBL) can serve as a template to fit an all-atom single-stranded RNA structure from the PDB. As an example, we show an alignment for phosphorous atoms from residues G${}_{61}$-U${}_{62}$-C${}_{63}$-G${}_{64}$ of 1J5E (Figure 1c, in magenta) to phosphorous atoms 3–6 in the vRNA trace of 4BBL. The sugar and nucleobase groups for U${}_{62}$ serve as an initial model of vRNA residue 4. Repeating this procedure across the whole phosphorous atom trace produces an atomic-scale vRNA model (Figure 1d, in magenta). Since vRNA principally binds to NP through its sugar-phosphate backbone, very few bad contacts are observed between the nucleobases and NP molecules of the initial structure, making alignment of the sequence from strain A/Albany/3/1967 [19] relatively straightforward.

#### 3.1.2. Connecting NPs between RNP Helical and Circular Regions

**Figure 2:**
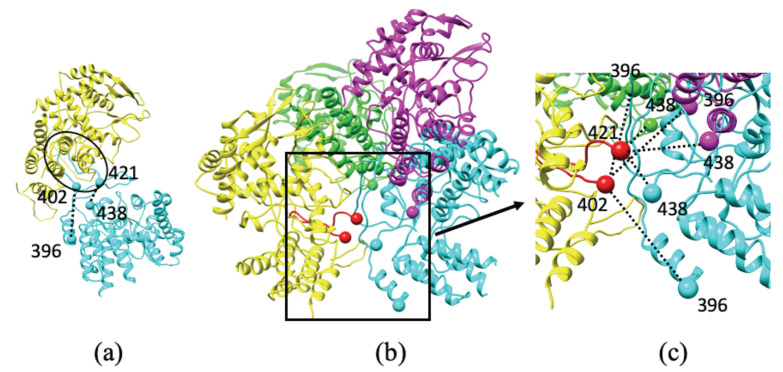
**NP dimer arrangements**. (**a**) Two interacting NP molecules from the trimer conformation (Protein Data Bank (PDB) accession code: 2IQH) are shown with the folded domain (residues 402-421) from one nucleoprotein (NP) (cyan) occupying the binding pocket of the second NP (yellow). Dotted lines between the folded domain and the main NP body are shown as a guide to the eye. (**b**) All three NP dimer arrangements are shown: trimer (cyan), circular (magenta), and helical (green) from PDB accession codes 2IQH, 2WFS, and 4BBL, respectively. The receiving NP (yellow), where the folded domain enters the binding pocket are superimposable in all three arrangements (red). (**c**) An enlarged view of the folded domain and the orientations of the trimer (cyan), circular (magenta), and helical (green) NPs shows the expected connectivity.

Based on structures determined using cryo-EM and X-ray crystallography, the RNP complex is shown to adopt a hairpin-like architecture, with two main classes of NP oligomerization: helical and circular suprastructures. In addition to these two types of filamentous suprastructures, NP is also observed to adopt a trimer arrangement (Figure 2a).

In all of these different arrangements, none of the oligomers involve direct contact between the main bodies of individual NP molecules [21,24,25,26,27]. Binding between two NP molecules depends on a small folded domain (residues 402–421) of one NP molecule that is projected out from the main globular protein body and into the binding pocket of a neighboring NP molecule. The orientation of the binding domain is dictated by two flexible linkers: (i) upstream residues 396–401, and (ii) downstream residues 422–438 (Figure 2). The short length of these linkers limits the number of orientations and conformations that NP–NP dimers can adopt and provides a rationale for the formation of the macromolecular hairpin complex. When a given orientation is repeated across multiple NPs, helical or circular oligomers are formed. We model the conformations of the linkers using methods similar to those previously employed for other protein complexes [15]. In brief, we select all atom protein fragments from the PDB that span the distance between the folded domain and the main NP body. Once a suitable backbone is identified and placed into the model, the correct sequence of amino acid side-chains is mutated.

**Figure 3:**
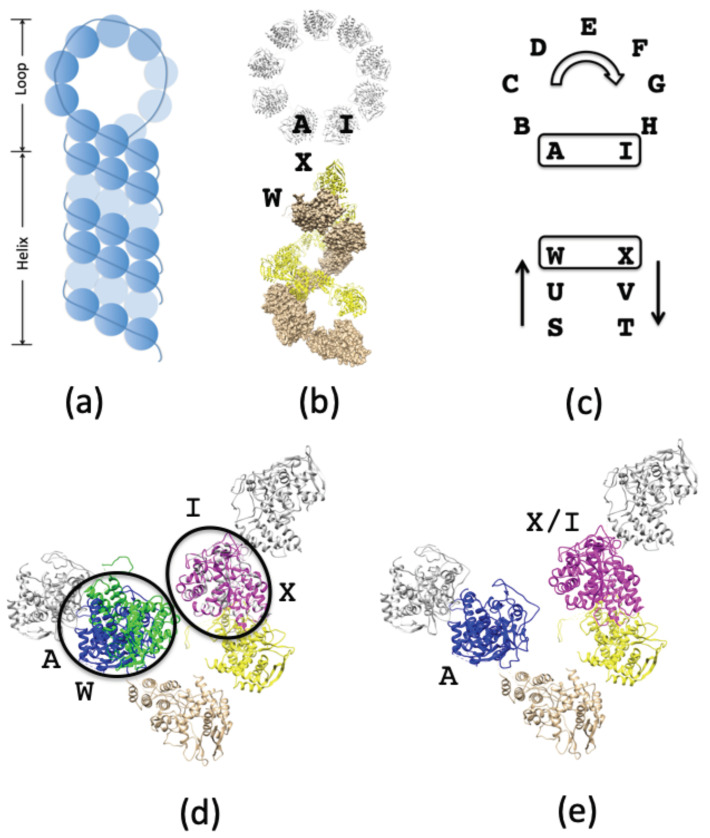
RNP complex. (**a**) The hairpin model of RNP is composed of two arrangements of NP oligomers (**b**): circular (gray) and helical (brown, yellow) suprastructures. (**c**) The two suprastructures (PDB accession codes: 2WFS, 4BBL) are mapped onto one another using two NPs in each structure (circular: A& I, helical: W& X). (**d**) After overlaying these NPs, one NP from each suprastructure is retained in the final model. (**e**) The close alignment of NP-X and NP-I means that either can be selected for the full RNP model. However, due to the orientations of the folded domain from NP-U and the binding pocket of NP-B, only NP-A can form a link between both NPs and unite the two suprastructures.

The full RNP hairpin [24,25,26] (Figure 3a) is composed of one long NP oligomer with both circular and helical suprastructures (Figure 3b) [16,42]. In order to combine the circular and helical suprastructures into a full hairpin assembly, we overlay two NP molecules from each suprastructure (Figure 3b,c). The overlayed NPs vary slightly in their orientations, necessitating careful realignments in order to ensure that the NP folded domains are projected into the binding pockets of the nearest neighbor NPs. The initial alignment shows that NP-I and NP-X overlay almost exactly (Figure 3d) while NP-A and NP-W both occupy the same space with divergent orientations. We analyze both NP molecules based on our knowledge of the folded domain in order to identify which molecule possesses the necessary orientation to serve as the bridge between NP-B and NP-U (Figure 3c). The folded domain of NP-W is unable to reach the binding pocket of NP-B, while the folded domain of NP-A is able to reach the binding pocket of NP-U while also accommodating the folded domain of NP-B into its binding pocket. Thus the structure of NP-A is selected to bridge the two regions (Figure 3e) and form the RNP hairpin into one continuous NP oligomer.

#### 3.1.3. Structure of RNA Loops between NP-Bound vRNA

**Figure 4:**
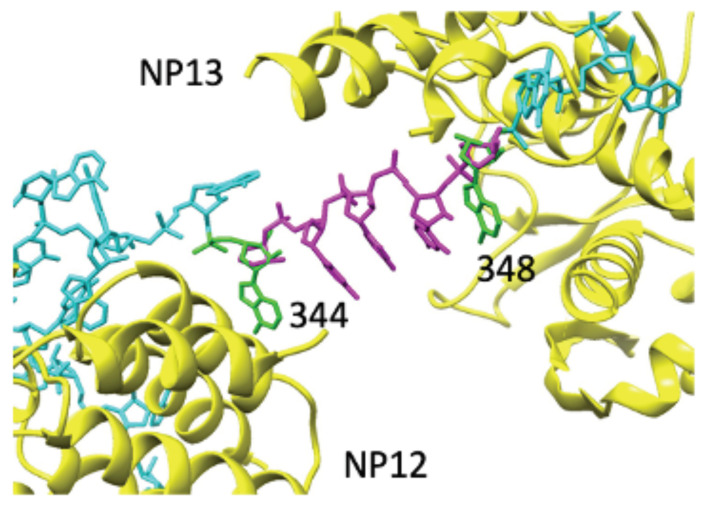
Bridging vRNA between two NP molecules. A five-residue loop (residues 230–234 from *T. thermophilus* 30S ribosomal subunit, PDB accession code: 1J5E) aligns with the vRNA nucleotides at the junction of the helical and circular suprastructures. The aligned residues of this RNA loop and the NP-bound vRNA (344 and 348) are shown in green, bridging residues are shown in magenta, and NP-associated vRNA residues are shown in cyan. The NP molecules are shown in yellow.

While the structures of vRNA on the surface of each NP can be generated from phosphorous atom coordinates, the geometry of the vRNA residues in the space between two NP molecules must be generated using a loop-modeling approach that satisfies two fundamental features: (i) aligning the terminal sugar moieties of the loop to the terminal sugar moieties in the vRNA already bound to the NP, and (ii) making sure that the modeled RNA loop does not encroach on the excluded volumes of the NPs, or the NP-bound vRNA. We identify RNA loops that satisfy both of these constraints by drawing structures from the PDB [9]. As an example, residues 230–234 from the *T. thermophilus* 30S ribosomal subunit (PDB accession code: 1J5E) are able to connect the vRNA bound to NP-12 in the helical suprastructure to the vRNA bound to NP-13 in the circular suprastructure (Figure 4) and form a continuous vRNA between the two NP molecules and thus both suprastructures of the RNP. The sugars on the ends of the chosen loop align with the sugars in the vRNA on both NP molecules and the vRNA loop is free of bad contacts with the rest of the RNP. This same procedure is repeated across the entire RNP structure to link the entire vRNA into a single gene segment.

#### 3.1.4. Model of NP-RNA Complex

According to experimentally-determined structures of RNP (EMDB accession code: emd_2205 and PDB accession code: 4BBL) each NP molecule in the helical suprastructure incorporates an average of 26 phosphorous atoms over its surface and through the space to its nearest neighbor NP. Thus, approximately 26 RNA residues coil around each NP molecule in the helical suprastructure. In the circular suprastructure (EMDB accession code: emd_1603 and PDB accession code: 2WFS) each NP molecule has an average of 20 associated phosphorous atoms. At the junction between the helical and circular suprastructures, the NP molecules show an average of 22 phosphorous atoms associated with each NP molecule. By fitting a segment of the vRNA NS1, the smallest gene, from *Influenza A*/Albany/3/1967 (855 residues, GenBank: CY020409.1) we find that 33 NP molecules can be arranged into the full hairpin structure, providing a comparison to the sequence and structure of the 8th RNP of *Influenza A* [43]. Our arrangement leaves approximately 60 RNA residues on the 5${}^{\prime }$ and 3${}^{\prime }$ ends of the vRNA to allow for POL-binding.

By assembling multiple known protein and RNA structures we thus generate an initial NP—vRNA hairpin structure that compactifies a full gene from the *Influenza A* genome.

### 3.2. Modeling the NP—vRNA—POL Complex

Binding of polymerase (POL) to the NP—vRNA complex is critical for the accurate assembly of the complete RNP macromolecule [17,18,21,24,25]. POL interacts directly with NP, and segments of vRNA can be accommodated into its binding cleft. Therefore our model must recapitulate the intermolecular interactions that govern such a structure.

#### 3.2.1. Selecting a Binding Mode for POL to the NP—RNA Complex

Loop-binding modes for POL are known [24], but the mechanistic view of POL transcribing vRNA into cRNA/mRNA from the 5${}^{\prime }$ to the 3${}^{\prime }$ end supports POL-binding on the helical terminus where the RNA termini are located [16,17,44].

#### 3.2.2. POL Binding to Double-Stranded RNA

Sequence-based studies of the *Influenza A* genome have identified two secondary structures that describe the 5${}^{\prime }$ and 3${}^{\prime }$ ends of the vRNA: a “corkscrew” [45] and a “panhandle” [46,47]. For viral genome replication, POL is able to recognize either structure.

**Figure 5:**
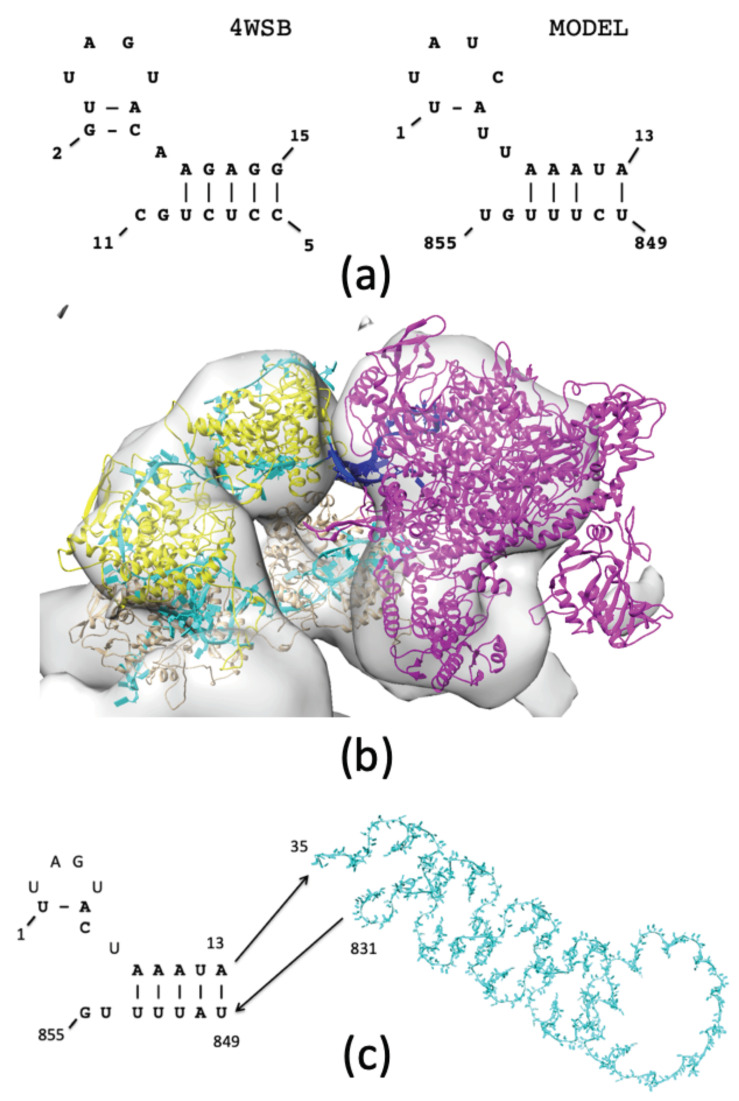
Modeling POL within the RNP complex. (**a**) The “half-corkscrew” secondary structure of vRNA (PDB accession code: 4WSB) is used to model POL-binding at the 3${}^{\prime }$ and 5${}^{\prime }$ termini. (**b**) Docking POL into the cryo-EM density map shows the vRNA promoter structure (blue) occupies the space between the NP—vRNA complex (yellow and cyan, respectively) and POL (magenta). A test of the goodness-of-fit for POL identifies 78.4% of the heavy atoms are retained within the outer contour of the EM density map of emd_2208 [16]. (**c**) Both ends of the vRNA in the “half-corkscrew” arrangement are joined with the rest of the vRNA at the residues shown.

Several new crystallographic structures of the whole *Influenza* polymerase complex have been determined, including one from bat *Influenza A* (PDB accession code: 4WSB), which includes the bound RNA promoter region [28]. In this particular structure, the promoter RNA sequences adopt a hybrid secondary structure called a “half-corkscrew” (Figure 5a) where the 5${}^{\prime }$ terminus of the vRNA forms a tetraloop, while the 3${}^{\prime }$ terminus ends in two hanging residues. This structure serves as a template for the promoter region of the vRNA from the *Influenza A*/Albany/3/1967 virus and anchors the POL to the rest of the NP—vRNA complex. Using the Chimera Fit-to-Map utility, a goodness-of-fit calculation identifies 78.4% of all heavy atoms within the outer EM contour in the final model.

#### 3.2.3. Structure of NP—vRNA—POL

The orientation of POL relative to the NP structure is obtained from cryo-EM data of the helical conformation of RNP (EMDB accession codes: emd_2205 and emd_2208); incorporating some of the same data used to model the RNP hairpin (Section 3.1.2). Using these data we can dock the POL structure (PDB accession code: 4WSB) into the same electron density map. In this arrangement, the RNA promoter within the binding site of POL faces the RNP, allowing the promoter to connect to the vRNA at the 5${}^{\prime }$ and 3${}^{\prime }$ termini (Figure 5b). The “half-corkscrew” from the promoter region completes the 5${}^{\prime }$ and 3${}^{\prime }$ termini (Figure 5c: 5${}^{\prime }$ residues (1–14) and 3${}^{\prime }$ residues (849–855)), while most of the other residues of vRNA (residues 35–831) are wrapped around the 33 NP molecules of the RNP complex. Using the secondary structure of the promoter as a template, the NS1 gene of the *Influenza A*/Albany/3/1967 viral sequence can be threaded directly into the promoter model. The connecting RNA loops (residues 15–34, 832–848) can then be modeled based on the phosphorous atom coordinates (see Section 3.1.1).

The final assembly of the NP—vRNA—POL complex incorporates a single NP oligomer with 33 molecules, a single vRNA chain (NS1 gene) with 855 residues, and a viral polymerase that encloses the “half-corkscrew” promoter at the 5${}^{\prime }$ and 3${}^{\prime }$ termini of the vRNA.

**Figure 6:**
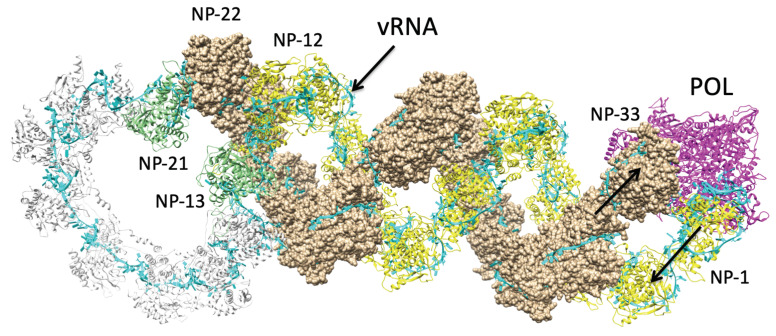
Structural model of the *Influenza A* NP–vRNA–polymerase (POL) complex. This model corresponds to the NS1 segment of the *Influenza A*/Albany/3/1967 RNP. Different NP oligomeric suprastructures are plotted as follows: helical NPs 1–12 (shown as yellow ribbons), circular NPs 13–21 (shown as gray ribbons), and helical NPs 22–33 (shown as tan calotte/space-filling). The vRNA (cyan) is plotted along the length of the RNP, and POL is shown in magenta.

Using an all-atom force field with previous applications to RNA and protein-RNA compounds [38,39,40] we conducted molecular dynamics simulations to resolve any residual bad contacts in the model and confirm any intermolecular salt-bridge contacts. A restraining potential was applied to the C${}_{\alpha }$ atoms in all amino acids, and to the phosphorous atoms in all nucleotides to prevent deformation of the hairpin structure while in a vacuum. This was found to be necessary, as initial simulations without these constraints caused the helical RNP region to extend and deviate from the confines of the electron microscopy envelope. After a 1000-step energy minimization, a 100-ps NVE ensemble simulation, and a follow-up 1000-step energy minimization, one final conformation (Figure 6) was produced. This final molecular model satisfies the bounds of the structure as defined by electron microscopy, maintains the atomic-scale structures of the circular and helical NP-oligomer regions, and most importantly, sheds light on multiple interactions between the many component molecules of RNP that play a role in the functional behavior of the RNP complex.

### 3.3. Determining Structure-Function Relations of the *Influenza A* RNP Complex

As we have described before, our model is able to describe the atomic coordinates of all protein and nucleic acid components of the complete RNP complex while simultaneously recapitulating the global structures defined by cryo-EM experiments. Thus we deliver unprecedented precision in terms of molecular geometry at scales where drug-binding and sequence mutations will have a demonstrable effect.

#### Direct Interactions between NP and POL

**Figure 7:**
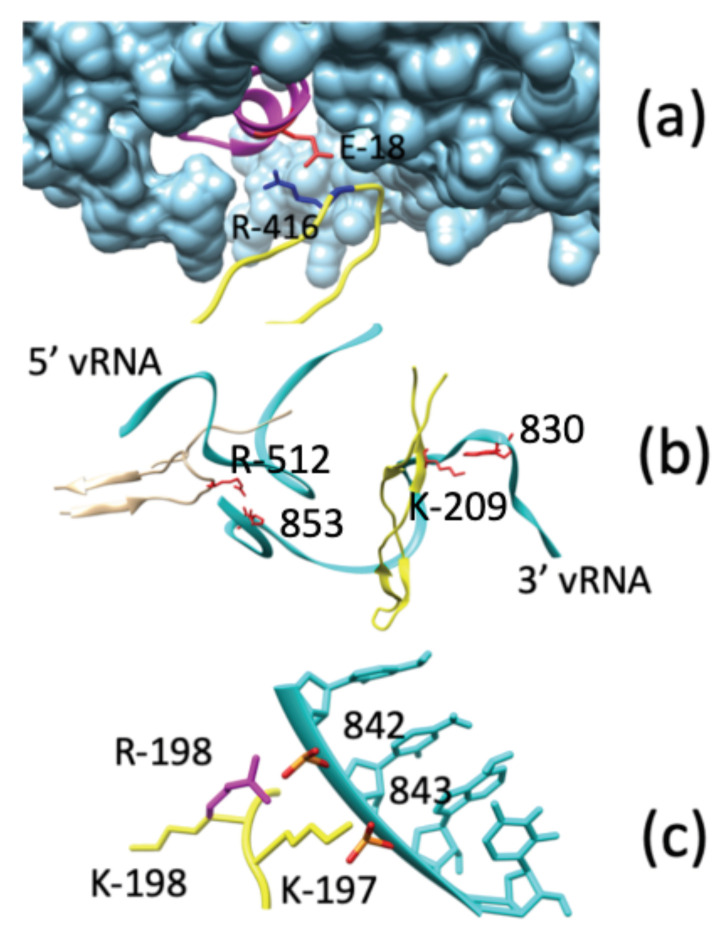
Interactions between POL and NP–vRNA. (**a**) The folded domain from NP-33 (residues 405–422, yellow) interacts with PB1 (cyan) and PB2 (magenta) of the polymerase. Residue R416 (blue) of NP-33, and E18 (red) of PB2 form a salt bridge. (**b**) Additional salt bridges are formed by the 3${}^{\prime }$ end of vRNA (cyan) to the polymerase subunits: A830 to K209 of PA (shown in yellow), and U853 to R512 within a hairpin structure of PB1 (shown in tan). (**c**) K198 of PB1 (yellow) points away from vRNA, while the K198R mutation (magenta) allows for a salt bridge to form with the phosphate group of C842 of the vRNA. The larger R198 side-chain thus causes an increase in POL-binding to vRNA.

An important feature in NP oligomerization is the binding of the folded domain of one NP-(*i*) to the binding pocket of its neighbor NP-(i+1). A salt bridge between R416 of NP-(*i*) and E339 of NP-(i+1) is an important feature of this interaction, and has been implicated as a drug target for *Influenza A* [33,48]. From our model (Figure 6), we find that the folded domain does indeed maintain this contact in the final optimized form, and that the terminal NP (NP-33, Figure 7) forms a similar interaction with the PB1 and PB2 components of the viral polymerase.

As shown in Figure 7a, residue R416 of NP-33 forms a salt bridge with E18 of PB2. Both E148 and E158 of PB1 are close enough to the folded domain to form salt bridges as well. This finding provides a structural explanation for the loss of POL activity in NP mutants like NP(R416A) [33]. Based on the similarity of NP-(PB1/PB2) interactions with those of NP–NP docking, we propose that the NP binding pocket on the polymerase might be a potential drug target for treating *Influenza* infections.

In our model, we also find that NP does not have direct contact with PA, which is consistent with observations from co-immunoprecipitation studies [49].

### 3.4. Direct Interactions between vRNA and POL

The interactions between vRNA and the subunits of POL in our RNP model offer additional insights into the functional behavior of RNP. Specifically, the interactions between vRNA and the PA and PB1 components of POL offer an explanation for how polymerase binding depends on multiple salt-bridge interactions. Our ability to compare mutations of one residue in PB1 (K198) even provides us with an explanation for the functional efficacy of the viral polymerase.

Two regions of PA (residues 370–380, and residues 506–519) form direct interactions with the vRNA (Figure 7b). Most are not sequence-specific, and include interactions between the main-chain of PA, or the sugar-phosphate backbone of vRNA. Only one sequence-specific, side-chain-to-nucleobase interaction occurs between PA (R512) and vRNA (U853).

For PB1, residues 177–214 form a β-hairpin loop containing multiple basic residues (Figure 7b). This hairpin has been hypothesized to interact directly with residues on the 3${}^{\prime }$ end of the vRNA [48], and in our model this is shown to be the case. Direct interactions occur with residues 828–855 of the 3${}^{\prime }$ end of vRNA. Similar to PA, a large number of salt bridges between the β-hairpin of PB1 and the vRNA are formed, suggesting a stable binding interaction between PB1 and vRNA. Only one sequence-specific, side-chain-to-nucleobase interaction occurs between the β-hairpin and vRNA: a salt bridge between K209 of PB1 and A830 of the vRNA. It is important to note that K209 is within one of the nuclear localization sequences of PB1 [50].

One other salt bridge between PB1 and vRNA has functional implications for polymerase activity: the R198 side-chain of PB1 and the phosphate backbone at residue C842 on vRNA. We find that the arginine side-chain has the requisite length to bring its guanidino group near enough to interact with the vRNA backbone, and is stabilized in our simulations. However, when the residue is mutated to lysine (R198K) the interaction is lost due to K198 not having enough bond lengths necessary to bridge the space between PB1 and C842 of vRNA (Figure 7c). The potential stabilization of POL-binding to vRNA through this salt bridge provides a structural explanation for the functional relevance of this residue in polymerase activity [51].

## 4. Discussion

While solving structures of biomolecular complexes in an experiment is critical, computational methods can also provide useful new insights into the mechanistic dependencies of large-scale biomolecular functionality. Our motif-matching fragment assembly (MMFA) method is able to leverage the vast amount of structural information from the PDB and EMDB as part of our structure determination process. Additional sequence information from GenBank [7] or the Influenza Virus Resource [19] can also be used to modify structures by mutating select residues, and allowing mutational assays to be investigated in silico. Macromolecular assembly, as we have shown here and in the past [11,13,14,15], is particularly well-suited for assembling large complexes in atomic detail, shedding light on how the component molecules are arranged relative to one another, and how particular interactions can stabilize these assemblies.

In this work, we have applied the aforementioned techniques to describe the structure of a single basic genomic unit of the *Influenza A* virus: the RNP complex. To the best of our knowledge, no other work has described the complete NP—vRNA—POL complex at this level of detail. It should be noted that there is no cryo-EM map of reasonable resolution for the full NP—vRNA—POL complex, so the structural validation done in this work has been conducted on certain components (i.e., docking of POL to helical terminus in Section 3.2.2).

The first challenge in generating the atomic model was determining atomic coordinates for the vRNA using available low-resolution cryo-EM information to guide the assembly of higher-resolution fragments. In this case, the data comes from the phosphorous atom trace of vRNA from the NP—vRNA complex (PDB accession code: 4BBL) [16]. Due to the lack of secondary structures in the vRNA, the approach that successfully modeled rRNA structures in our previous work [11] could not be applied here. Using one of the most general representations of all-atom experimental RNA structures (PDB accession code: 1J5E) a correlation was identified between nucleobase orientation and the positions of the phosphorous atoms. Based on this simple correlation, an all-atom model of the vRNA structure was generated from the coordinates of phosphorous atoms alone. This algorithm is general purpose and can be used to model other single-stranded nucleic acid molecules based on similar low-resolution phosphorous atom coordinates.

The second big challenge was assembling the two higher-order NP suprastructures (circular and helical) into the hairpin conformation of RNP. Using our knowledge of oligomeric structures of NPs, the requisite orientations of the folded domains (residues 402–421) relative to the binding pockets of neighboring NP monomers, and our knowledge of the flexibility of biopolymer loops, we modeled the loops responsible for orienting the folded domains throughout the entire RNP complex. Thus we formed one continuous NP oligomer with helical and circular supramolecular structures that describes the hairpin conformation of RNP and is consistent with observed cryo-EM structures.

*Influenza* polymerase (POL) binds to both 5${}^{\prime }$ and 3${}^{\prime }$ ends of the vRNA when the latter forms either a “corkscrew” or “panhandle” secondary structure [45,46,47]. Using the crystal structure of the bat *Influenza A* polymerase-vRNA complex as a template we modeled a “half-corkscrew” structure of vRNA and successfully docked POL complex to the NP—vRNA complex using a low-resolution (22.1 Å) cryo-EM map of a POL-NP complex as a guide. Our loop modeling techniques then fill in the remaining gaps in the vRNA structure between the POL and NP.

Following simple energy minimizations and an NVE simulation to refine our model, we identified multiple salt-bridge interactions involving side-chain and nucleobase groups that are stabilized in our final RNP model. Importantly, these interactions provide valuable insight into the functional behaviors of the RNP that are only apparent after our final assembly is complete.

At atomic-scale we find that NP-NP oligomerization is stabilized by a salt bridge between NP-(i)(R416) and NP-(i+1)(E339), as expected from viral target studies [33,48]. Our model shows that a similar interaction forms between the terminal NP and the polymerase: NP-33(R416) to PB2(E18). Additional salt bridges between NP-33(R416) and PB1(E148) or PB1(E158) are also possible. Our findings support experimental mutational analyses showing that viral polymerase activity is not supported in NP mutants such as R416A [33].

We find that the vRNA contributes further to RNP stability with salt bridges between PB1(R198) and vRNA(-PO${}_{4}$- of C842). A cRNA-orientated minigenome assay of a PB1 mutant (K198R) confirms this functional basis of this structural arrangement based on a six-fold increase in polymerase activity [51]. The interaction between PB1(K209) and vRNA(A830) is the only sequence-specific interaction between PB1 and vRNA, and is part of the nuclear localization signal 2 (NLS2) of PB1. Mutational analyses have shown that this site has little effect on nuclear localization [50], however, follow-up mutational analyses show that mutating this residue reduces viral rescue 4-fold by inhibiting binding to the beta-importin Ran-binding protein 5 (RanBP5) [52]. These results lend further credence to the value of our model as a tool to validate a variety of experimental results.

In addition to providing structural evidence that supports the results of various experiments involving protein mutations, our findings also call into question the effects of the vRNA sequence on POL-binding. Such specific interactions as we find have never been observed or discussed before, meaning that our methods could be used to seek out additional protein/nucleic-acid interactions for other viral genome segments, or offer the possibility of identifying novel drug targets for inhibition of *Influenza*. These results could open the door to future studies in viral protein mutagenesis or viral sequence analyses to assess the efficacy of POL-binding and activity.

## 5. Conclusions

One of the biggest challenges for structural modeling in the coming decades will be solving macromolecular structures of large biomolecular complexes, such as the genomic segments from living systems.

The *Influenza* virus has eight fragmented genome pieces, and each fragment is a stand-alone molecular machine that carries out functions related to the viral life cycle. Our progress towards understanding how the virus functions through its life cycle is hindered by the lack of a detailed structure of an intact *Influenza* NP-vRNA-POL complex. Through multiple efforts by many laboratories and scientists, sequence and structural information of this complex at different levels of detail, resolution and completeness have become available, but have never before been consolidated to model a complete RNP complex in atomic detail.

By combining these data with in silico MMFA modeling approaches we have developed a model of a single *Influenza* RNP complex and its associated polymerase in atomic detail—a crucial advance to complement many years and multiple studies of RNP structure determination through cryo-EM [16,17,18], and X-ray crystallography [21,28,42]. The model leads us to a deeper and more refined view of the structure-function relationships of the RNP complex, which can be used to guide future studies of *Influenza*. This work is an important step toward modeling RNP complexes at various stages in the viral life cycle [18], and toward modeling a complete viral particle in atomic detail. These advances will serve to influence and inform further research in viral biophysics from atomic-scale to the scale of full viruses.

## Figures and Tables

**Table 1:** Modeling individual components of ribonucleoprotein (RNP). Multiple sequence and structural resources are employed as part of generating the full RNP complex. The individual target components of the RNP model, and the data sources and content needed to produce each target, are provided herein.

RNP Model Target	Data Source(s)	Data Content	Critical Components to Model
vRNA (helical domain)	PDB_ID: 4BBL PDB RNA fragments	Phosphorous atoms in RNP helical region	All-atom vRNA
vRNA (sequence)	Influenza Virus Resource [19], GenBank [7], PDB [9]	Primary sequence of vRNA	All-atom vRNA structure with a target Influenza A sequence
NP (helical domain)	PDB_ID: 4BBL, EMDB_ID: emd_2205	NPs in helical conformation	NP folded domain orientation
NP (circular domain)	PDB_ID: 2WFS, EMDB_ID: emd_1603	NPs in hairpin loop conformation	NP folded domain orientation
POL + vRNA (promoter)	PDB_ID: 4WSB	POL with docked RNA promoter	POL docked with “Half-corkscrew” promoter conformation
NP + RNA + POL	EMDB_ID: emd_2208	vRNA + NP + POL targets	POL docking to RNP

## Abbreviations

The following abbreviations are used in this manuscript:

MDPI	Multidisciplinary Digital Publishing Institute
DOAJ	Directory of open access journals
RNP	ribonucleoprotein
RNA	ribonucleic acid
POL	polymerase
MMFA	motif-matching fragment assembly
